# Development and pilot of a tool evaluating community-engaged group processes and community-centered impact for institutional level advisory boards

**DOI:** 10.1017/cts.2025.10177

**Published:** 2025-10-28

**Authors:** Michele Allen, Yasamin Graff, Caroline Carlin, Antonia Apolinario-Wilcoxon, Paulette Baukol, Kristin Boman, LaPrincess Brewer, Roli Dwivedi, Milton Eder, Susan Gust, Mikow Hang, Walter Novillo, Luis Ortega, Shannon Pergament, Chris Pulley, Rebecca Shirley, Sida Ly-Xiong

**Affiliations:** 1 Department of Family Medicine and Community Health, University of Minnesota, Minneapolis, MN, USA; 2 Clinical and Translational Science Institute, University of Minnesota, Minneapolis, MN, USA; 3 Carlson School of Management, University of Minnesota, Minneapolis, MN, USA; 4 Equity Strategies LLC, Minneapolis, MN, USA; 5 Hazelden Betty Ford Foundation, Center City, MN, USA; 6 Department of Cardiovascular Medicine, Mayo Clinic, Rochester, MN, USA; 7 Center for Clinical and Translational Science, Mayo Clinic, Rochester, MN, USA; 8 SoLaHmo Partnership for Health & Wellness, Minneapolis, MN, USA; 9 Ly-Xiong Enterprises, Madison, WI, USA

**Keywords:** Community engagement, mixed methods, participatory group process, institutional change, evaluation

## Abstract

**Introduction:**

While evaluation approaches for community-academic research groups are established, few tools exist for academic institutional advisory groups across multi-core centers and research, education, and clinical care missions. Institutional advisory group evaluation should consider group processes and their impact on community-centered outcomes. This study describes the community-engaged development of a mixed-method evaluation approach to address this gap and presents pilot outcomes across an NIH-funded center.

**Methods:**

We utilized a Community of Practice model to co-develop a survey with 14 community and academic representatives of four advisory groups. The final survey included five categories of group process and four categories of outcomes. Storytelling sessions with community partners explored areas where the survey identified discrepancies in perspectives between community and academic team members, as well as areas with lower scores.

**Results:**

Nine community and 14 academic (staff and faculty) partners completed the survey. Respondents positively assessed group process outcomes (shared values, leadership, community-centeredness, and decision-making), and slightly less positive assessments of institutional outcomes. Storytelling sessions confirmed the overall satisfaction of community partners but highlighted actionable concerns within power-sharing, decision-making, funding equity, and trust-building.

**Conclusions:**

The results of this equity-centered evaluation suggest the utility and importance of participatory, mixed-methods approaches to evaluating community-academic institutional advisory groups.

## Introduction

Community engagement (CE) has been a cornerstone of translational science and research on health disparities for over twenty years. Academic entities, including multi-core centers and health-focused institutes, particularly those funded by the National Institutes of Health (NIH), are increasingly asked to include community partners and other stakeholders in their structure to develop sustainable and effective programs addressing health equity [1,2]. More recently, CE has been promoted to enhance the broader connection between academic health centers and the communities they serve, extending beyond research to include teaching and clinical care missions [3].

Evaluations of study-specific community-academic research partnerships have generally found that the deeper the perceived quality of group partnership processes, the stronger and broader the short- and long-term impact and outcomes [4–7]. The increasingly validated surveys used to evaluate partnership processes (e.g., leadership structure, decision-making, communication) and structural factors (e.g., who is involved, what is their role), have deepened the science of CE by identifying how investment in group function contributes to process and ultimately to research outcomes [8]. For example, evaluations indicate that participatory processes, such as shared power and decision-making between community and academic collaborators, promote equity, and foster trust in the research enterprise [2,9,10]. Additionally, evaluation approaches have identified that strong, authentic, and long-lasting partnerships help ensure that research products are culturally sensitive and community-relevant, and enhance their quality, efficacy, and sustainability [9,11]. This research has advanced the science of CE, specifically regarding the relationship between community involvement and research outcomes.

In addition to assessing community-centered outcomes, a key purpose of CE evaluation is to identify areas for training and support to deepen participatory group processes and increase the impact of CE academic entities [12]. Equity-centered, community-driven evaluation requires mixed-methods approaches that incorporate both structured and narrative-based inquiry [13]. Furthermore, when communities are involved in shaping the evaluation process, research becomes both a means and a product of relationship-building, cultural alignment, and accountability [14]. Inclusive methods are particularly effective in surfacing structural inequities and power differentials, offering not only reflection but also a pathway toward actionable change [15].

While the number of tools and approaches, including validated surveys, are increasing, fewer tools are available for evaluating the work of multi-core centers or across institutional missions. The expanding scope of CE suggests a need for evaluation approaches that utilize a community-engaged evaluation approach to assess the quality and community-centered impact of advisory bodies across institutional missions and outcomes and provide means for improvement. A recent survey was developed specifically to assess academic health system-level institutional facilitators and barriers to community-engaged research [16]. However, this survey did not address CE across education and clinical care missions. Furthermore, it was designed primarily as a quantitative assessment of institutions, with a focus on identifying actionable narratives through qualitative assessment.

While we expect the participatory processes within CE research and larger academic entities to be similar, the outcomes may be different, given that the work of academic entities may be broader than the focused work of a CE research project. For example, community members are often motivated to participate on advisory boards because they perceive opportunities to improve how institutions function or to impact policies or procedures related to the health and well-being of their communities [17]. Therefore, evaluation approaches must consider how the academic entity is structured and operates in terms of the experiences of community partners to assure mutually beneficial and equitable processes, and community-centered outcomes.

Within current frameworks for CE evaluation, there is a gap in tools that are tailored to the specific contexts, objectives, and challenges of academic entities that consider ways that the structure, organization, and processes of the entity contribute to, or undermine engagement with community perspectives, priorities, and power-sharing. This study describes the development and piloting of a mixed-methods tool, including an adapted survey and follow-up community inquiry to evaluate CE partnerships at multiple levels and across missions (research center and project; education; and clinical care). Consistent with our CE approach, we leveraged the Community of Practice (CoP) infrastructure established within the CE core of a National Institute of Minority Health and Health Disparities-funded P50 center through a collaborative process that included representatives from multiple community-academic advisory boards (Boards). The tool is intended to evaluate the advisory and joint decision-making capacity that community members experience within these groups and their perceptions of the groups’ success in achieving community-centered outcomes. It was designed to assess engagement and examine relationships, power dynamics, and pathways for lasting institutional change. This evaluation tool is intended to be used by advisory groups across academic health centers so they can understand and improve how community-academic partnerships function and achieve community-centered outcomes that create opportunities for growth.

## Methods

The University of Minnesota Institutional Review Board determined that this study does not constitute human subjects research.

### Procedures

Using a CoP approach, we brought together community and academic representatives of multiple Boards to create an evaluation tool. Key characteristics of the CoP approach include developing a shared purpose, fostering shared learning, building trust, promoting mutual engagement, and committing to the process [18,19]. CoPs can have different goals and be structured and organized differently, but all include three main components: domain, community, and practice [18,19]. Domain is the topic of the CoP and the group’s focus. Community is who needs to be part of the group. Practice is what the group will work on together. For this CoP, the domain topic was evaluating Boards, and the practice was developing an evaluation tool. For community, this was an invite-only CoP, meaning participants were invited due to their experience as a member of an institutional board focusing on clinical care, research, or education initiatives within academic health centers, or expertise in crafting evaluation tools. Equal numbers of academic and community partners were invited to participate. Academic members came from multiple departments and programs. Community members represented various advisory boards across the Medical School. Final membership is described in Table 1.

**Table 1.** Characteristics of community of practice participants*

	N	%
Group		
A Health equity program	5	31%
B NIH-funded center	8	50%
C Department-level clinical and educational efforts	2	12%
D Practice-based research networks	1	6%
E Total number of surveys submitted across groups	16	100%
Role		
Community partner	7	50%
Academic faculty	3	21%
Academic staff	4	29%
Total number of unique respondents	14	100%

* The discrepancy between the number of surveys and the number of unique respondents is due to two participants being representatives of multiple groups. They completed the survey for each group to which they belong. They hold the same role for each group.

The CoP series consisted of seven meetings from March to October 2023. The group first developed a shared definition of the work goals. To create a common understanding of community-engaged practices, the group identified the positions of the various participating Boards on the continuum of CE [20] and reached a consensus on the ideal placement, which was the “collaborate” categorization. While the group identified an aspirational placement of “shared leadership within the group,” they recognized institutional constraints on full co-leadership, given their structures. The group determined that “collaborate” was to be measured where the bidirectional partnership is characterized by trust, and all partners are active in all discussions and group decisions [20].

The academic lead and staff conducted a literature review and presented results to the group. The group determined that no existing evaluation tool met the intended purpose of evaluating group processes and outcomes across institutional advisory boards. The group reviewed established evaluation tools for partnership processes within community-engaged research projects and found that, while the themes addressed were similar, they would need to be adapted. The tools reviewed included the Goodman Quantitative CE Measure [6], Engage for Equity CE Survey [4,5], and the Trust Typology Model [7]. We then worked together across multiple meetings to review and adapt the questions to be relevant for institutional advisory boards. Where existing surveys did not address specific prioritized topics, the group developed new questions based on their experiences within Boards and knowledge of participatory research. In the end, we created a quantitative 35-question study addressing five areas of quality community-academic group process (Depth of Involvement, Shared Values, Leadership Practices, Community-Centeredness, and Decision-making), and community-centered outcomes for clinical care, research, and education. See Table 2 for all questions. Branching logic differentiated outcomes across boards focused on each area. A prior tool’s use of a 7-point Likert scale (7) and community preference for more response options to some questions resulted in use of both 5 and 7 option Likert scales as specified in Table 2.

**Table 2.** Final survey developed by community of practice

Survey categories	Questions/response categories
General survey questions for all participants	
Member attendance and involvement****	How long have you been a member of the group?**** Less than 1 year; 1-2 years; 3-7 years; 8+ years
	What is the total number of meetings you attended with the group in the past year?*** Open number response
	My involvement influenced the group to be more responsive to the community.* ^#^Agreement Likert scale
Shared partnership values, capacity, and synergy*	Our group showed respect towards one another.* ^#^Agreement Likert scale
	Our group listened to each other.* ^#^Agreement Likert scale
	The community members of our group had the knowledge, skills, and confidence to interact effectively with the academic partners.* ^#^Agreement Likert scale
	The academic members of our group had the knowledge, skills, and confidence to interact effectively with the community partners.* ^#^Agreement Likert scale
	Our group had a clear and shared understanding of our goals, priorities, and strategies.* ^#^Agreement Likert scale
	Our group had discussions about our role in promoting strategies to address social and health equity.* ^#^Agreement Likert scale
	When conflicts occurred, members of our group worked together to resolve them.* ^#^Agreement Likert scale
	Even when we didn’t have total agreement, our group reached a general consensus that we all accepted.* ^#^Agreement Likert scale
Collaborative leadership*	Our group fostered a space where all members are leaders.**** ^$^Quality scale
	Our group recognized different forms of leadership within the group.**** ^$^Quality scale
Community- Centeredness****	Our group focused on topics important to our communities.** ^#^Agreement Likert scale
	Our group promoted equity by focusing on personal, social, economic, and cultural factors that influence health behaviors and health status.** ^#^Agreement Likert scale
	Our group was responsive to the ways in which institutions have impacted the community.* ^#^Agreement Likert scale
	Our group was responsive to community histories and knowledge.* ^#^Agreement Likert scale
	Our group attempted to learn and incorporate the opinions and perspectives of the broader community into its decisions and programs.**** ^#^Agreement Likert scale
Partnership trust typology*	What primary type of trust do you think your group has now?* Trust deficit: partners do not trust each other* Neutral: partners are still getting to know each other; there is neither trust nor mistrust;*** Role-based: based on partners’ title or role with limited or no direct interaction prior to this project;*** Functional: partners are working together for a specific purpose or timeframe;* Proxy: partners are trusted because someone who is trusted invited them;* Reflective: trust which allows for mistakes and where differences can be talked about and resolved*
Participatory decision-making*	How often did you feel comfortable with the way decisions were made in the group?* +Frequency scale
	How often did you feel that your opinion was taken into consideration by other group members?* +Frequency scale
	How often did you feel pressured to go along with decisions of the group even though you might not have agreed?* +Frequency scale
	How often were all group members’ ideas and opinions taken into consideration when decisions were made by the group?* +Frequency scale
	How often did you feel that all members had the power to promote decisions that would benefit the communities we worked with?*+Frequency scale
Branching: Outcome questions for clinic-based advisory groups	
Changes in clinic procedures, policies, or practices*	Over the past year, this group produced better coordination between the clinic and/or healthcare system and patient and community needs.* ^#^Agreement Likert scale
	Over the past year, this group produced changes in the clinic and/or the healthcare system organization’s approach to important health issues in the community.* ^#^Agreement Likert scale
	Over the past year, this group produced changes in the clinic and/or healthcare system policies, procedures or practices.* ^#^Agreement Likert scale
	Over the past year, this group produced sustainable changes in the clinic and/or healthcare system’s approach to important health issues in the community.**** ^#^Agreement Likert scale
	Over the past year, this group produced more explicit equitable and anti-racist practices.* ^#^Agreement Likert scale
	Over the past year, this group produced approaches to integrate community perspectives into institutional decision-making.*^#^Agreement Likert scale
	Over the past year, this group produced activities that engage community in identifying and addressing their priority health issues.*^#^Agreement Likert scale
	Over the past year, this group promoted cultural identities or pride.* ^#^Agreement Likert scale
	Over the past year, this group impacted patient experience.**** ^#^Agreement Likert scale
Branching: outcome questions for advisory groups with education or research initiatives	
Institutional policies, procedures, or practices changes*	Over the past year, this group produced better coordination between the institution and community groups.*^#^Agreement Likert scale
	Over the past year, this group produced changes in the institution’s approach to important health issues in the community.* ^#^Agreement Likert scale
	Over the past year, this group produced changes in institutional policies, procedures, or practices.*^#^Agreement Likert scale
	Over the past year, this group produced sustainable changes in the organization’s approach to important health issues in the community.* ^#^Agreement Likert scale
	Over the past year, this group produced more explicit equitable and anti-racist institutional practices.**** ^#^Agreement Likert scale
Institutional actions integrated into community*	Over the past year, this group produced approaches to integrate community perspectives into institutional decision-making.* ^#^Agreement Likert scale
	Over the past year, this group produced projects that engaged the community in identifying and addressing their priority health issues.* ^#^Agreement Likert scale
	Over the past year, this group produced positive action steps towards the co-development of community practices, programs, or policies.* ^#^Agreement Likert scale
	Over the past year, this group produced approaches to share lessons learned with other community/academic groups.**** ^#^Agreement Likert scale
Social transformation*	Over the past year, did this group produce reinforced cultural identity or pride?* ^#^Agreement Likert scale
	Over the past year, did this group produce broad social impacts?* ^#^Agreement Likert scale

* Adapted from Engage for Equity Community Engagement Survey [4,5].

** Adapted from Goodman Quantitative Community Engagement Measure [6].

*** Adapted from the Trust Typology Model [7].

**** Questions were developed by the members of the Community of Practice.

# Agreement Likert scale: Completely disagree; Mostly disagree; Slightly disagree; Neither agree or disagree; Slightly agree; Mostly agree; Completely agree.

^$^Quality Likert scale: Poor; Fair; Good; Very good; Excellent.

+Frequency Likert scale: Never; Rarely; Sometimes; Often; Always.

We piloted the survey within a large, NIH-funded center that included a community-academic steering committee, CE core co-led by a community-academic coalition, and three studies that included community-academic advisory groups. One of the groups represented a long-standing community-academic research partnership, while others came together for the current study.

The survey was emailed to the 59 total community and academic members of the five advisory groups via REDCap [21]. Based on survey results, qualitative questions were developed by community evaluator SLX and reviewed by the team to gain clarification and dig deeper into areas of potential growth or discrepancies between respondents. Consistent with our intention to identify specific examples for improvement from community members, we conducted three storytelling (focus group) sessions . Community members who completed the survey were invited by email to participate in a storytelling session. Eight community members representing five partnerships across C2DREAM participated. Sample questions included: (1) How effectively does your group “center” community perspectives into its processes; (2) How do you perceive power dynamics within the group and/or between academic and community partners; and, (3) Do you feel that decision-making processes within your group are collaborative? Each storytelling session was conducted virtually and recorded. Notes were taken during the group, and recordings were transcribed for clarity. With the storytelling sessions we intended to capture actionable qualitative results on areas of group process improvement and specific steps institutions can take to strengthen advisory board engagement.

### Analysis

Scores were developed from Likert responses within each section of the quantitative survey. This was done by dividing the response by the maximum 5- or 7-point response to normalize scale, with reverse coding as needed, and then averaging across responses within the survey section, creating a score within the range 0 to 1. As an initial check for internal consistency, Cronbach’s alphas were calculated for each group process scale, using the normalized scores. Though this survey was not intentionally powered for statistical assessment, bivariate statistical significance of the associations between Likert responses from individual questions and respondent characteristics were assessed using Pearson Chi-Square tests. Similarly, bivariate significance of the associations between continuous normalized mean scores and respondent characteristics were assessed using ANOVA tests. Given the multiplicity of tests, a p-value of 0.01 was selected to identify significant associations highlighted here. We also considered tests where a p-value was less than 0.10 as a marginally significant association for individual items to consider for further exploration through storytelling group questions.

The qualitative portion of our evaluation utilized participatory evaluation methods in the design and implementation of the evaluation [22,23]. The team co-created questions utilized in community storytelling sessions to further explore the meaning of the quantitative results, particularly in survey topic areas that highlighted different perceptions across respondent groups [24]. Storytelling sessions were conducted via a meeting platform and verbatim notes were taken by two individuals and combined. Community evaluator SLX analyzed the qualitative data from the storytelling sessions using a deductive analytic approach, summarized the results, and identified themes within each topic area.

## Results

### Quantitative survey results

For the pilot survey, of the 59 surveys sent out, 23 were completed, representing 22 unique respondents (one individual completed a survey representing membership on two boards). The responses are weighted toward those representing two of the five advisory groups (Table 3). Both academic and community partner roles are represented among respondents (39% community respondents). About half of the respondents have three or more years of experience with their group, with slightly more than half having participated in more than six meetings over the last 12 months. Results of the trust typology assessment indicate that approximately 70% of respondents described trust at the proxy or reflective level (see Table 2 for typology).

**Table 3.** Characteristics of pilot survey respondents

	N	%
Total number of respondents	23	100%
Advisory group		
A steering committee	3	13%
B Community engagement coalition	8	35%
D Study advisory board	2	9%
E Study advisory board	3	13%
F Study advisory board	7	30%
Role		
Community partner	9	39%
Academic faculty	9	39%
Academic staff	5	22%
Tenure on board		
Less than 1 year	1	4%
1–2 years	10	43%
3–7 years	8	35%
8 or more years	4	17%
Total number of meetings attended (past 12 months)		
0–3 meetings	7	30%
4–6 meetings	5	22%
7–49 meetings	7	30%
50+ meetings	4	17%

Respondents’ evaluation of the four areas of community-engaged group processes were positive, with means ranging between 0.848 (SD = 0.112) for shared leadership, and 0.894 (SD = 0.151) for shared values, suggesting that they largely strongly agreed with the positive evaluation of how the group operated (Table 4). Cronbach’s alphas for each group process scale were high (above 0.90) except for leadership which was (0.694) suggesting moderate to strong internal consistency of these measures. Respondent’s assessment of research-focused outcomes (See Table 2 for questions) was slightly less positive, (mean = 0.787, SD = 0.142) suggesting there was less agreement on the impact of the group on these outcomes.

**Table 4.** Distribution of likert responses scores* by survey section

	Mean	Standard deviation	Quartiles			Cronbach alpha
			25th	50th	75th	
Shared values	0.894	0.112	0.857	0.929	0.982	0.924
Leadership	0.848	0.151	0.714	0.857	1.000	0.929
Community-Centeredness	0.871	0.132	0.821	0.929	0.964	0.901
Decision-making	0.853	0.105	0.766	0.880	0.960	0.694
Research-focused group outcomes	0.787	0.142	0.714	0.831	0.922	0.942

* Likert responses were expressed as a fraction of the maximum response (5- or 7-point scale), reverse coding as needed so that increasing score represents positive perception, and then averaged across questions within the section to produce a score.

We also assessed bivariate associations between individual survey items and variables identifying advisory group, respondent role (community, faculty, staff), tenure, and frequency of involvement. Only one statistically significant association emerged. A stronger belief in the group’s ability to resolve conflicts was associated with an increase in the number of meetings (*p* = 0.004). None of those who attended 0–3 meetings agreed that the group worked together to resolve conflicts when they occurred. In contrast, 57.2% of those who attended 7–49 meetings and 66.7% of those who had attended 50 or more meetings in the last year completely agreed.

Outcomes with marginal significance (p-value of ≤ 0.10), considered for further exploration through storytelling sessions, included those associated with a specific advisory group (questions regarding shared partnership values, and research outcomes), respondent role (staff may have greater comfort in group decision-making, and community may feel pressured to conform in decision-making), and frequency of involvement (questions regarding shared partnership values, collaborative decision-making, and education and research initiatives).

### Qualitative action-oriented results

A total of eight individuals, representing five partnerships, participated in three story telling sessions. Qualitative results indicated that participants widely agreed that the center prioritized CE through structured mechanisms such as advisory councils, steering committees, and training programs. Community partners indicated that their impact was greater when they actively shaped decisions rather than just providing input. One participant shared how a community-driven idea for a cardiovascular health app became a reality, demonstrating that when institutions listen to communities, engagement leads to real outcomes. Another example highlighted research team responsiveness when they simplified presentations, added cultural considerations, and made materials more practical. Community members described the long-term value of including their expertise. One said, *We’re not just here to advise – we bring solutions. When we are truly included, the work is better, and the community benefits.*

Five thematic, action-oriented outcomes emerged (Table 5), identifying barriers to engagement and cultural nuances. Stories highlighted institutional dominance over final funding, decision-making, and research priorities as barriers. They emphasized the need for more equitable payment mechanisms to minimize financial strain on community partners and for hiring community members in research roles rather than relying solely on stipends. Additionally, they identified a need for greater transparency in decision-making to ensure advisory board contributions are fully acted upon.

**Table 5.** Institutional recommendations for strengthening equity-centered practices

Recommendation to center community and equity	Findings from qualitative data	Sample actionable steps for institutional change
**Strengthen long-term trust-building by deepening researcher involvement in unstructured community spaces.**	- While trust scores were high in surveys, storytelling sessions emphasized that trust is not a fixed achievement but an ongoing process requiring continuous engagement. - Community members noted that researchers are visible in structured meetings but often absent from community-driven spaces, making engagement feel transactional rather than relational. - Existing trust tends to be uneven, stronger in long-standing relationships but weaker where new partnerships are forming.	- Shift from a meeting-based engagement model to a sustained relational model by encouraging researchers to participate in community spaces outside of institutional structures. - Establish long-term engagement plans that extend beyond the life cycle of specific grants. - Increase opportunities for co- designed engagement strategies that are flexible and responsive to community needs.
**Increase funding equity to prevent financial burdens on community organizations.**	- Community partners face delays in reimbursement and financial instability when required to front costs for research participation. - Honorariums acknowledge contributions but do not provide sustainable financial support for long-term engagement. - Structural disparities in funding make it harder for community organizations to commit to research partnerships consistently.	- Transition from reimbursement models to direct funding mechanisms that provide upfront financial support to community organizations. - Create equitable compensation structures that ensure community partners are financially supported similarly to academics. - Build sustainable funding pipelines to support community participation beyond short-term project cycles.
**Formalize decision-making transparency to ensure all partners remain informed.**	- Survey results suggested perceptions of shared leadership, yet storytelling group participants reported unclear decision-making processes where changes sometimes occurred without community input. - Community members highlighted frustration with limited follow-up on board recommendations, making participation feel tokenistic. - Decision-making remains institutionally controlled, despite participatory rhetoric.	- Implement structured decision-making documentation that tracks community recommendations and institutional responses. - Transparency of reporting mechanisms are required where advisory board contributions are visibly reflected in institutional decisions. - Establish formalized decision-sharing processes to shift from consultative to co-governance models.
**Hire more community members in research roles, valuing lived experience as expertise.**	- Community partners expressed concern that academic credentials are prioritized over lived experience when hiring for research roles. - Reliance on advisory board participation rather than formal hiring pathways limits the long-term role of community members in shaping research. - Power dynamics remain inequitable when decision-making is concentrated among institutionally affiliated leads.	- Establish community research fellowships to embed non-academic experts into institutional research teams. - Develop hiring policies that recognize lived experience as a research qualification. - Increase leadership pathways for community members, ensuring they have opportunities to transition into decision-making roles.
**Expand culturally appropriate communication methods, such as infographics, videos, and multilingual resources.**	- Community partners reported that institutional communication styles are often inaccessible, overly academic, or overwhelming. - Email-based communication was seen as excessive, with unclear distinctions between informational updates and action-driven messages. - Participants preferred visual, oral, and multilingual communication tools to improve engagement.	- Develop multimodal communication strategies, including infographics, short videos, and community-led storytelling. - Ensure all materials are translated into relevant languages to reflect the linguistic diversity of participating communities. - Implement streamlined communication processes that distinguish actionable requests from general information sharing.

Participants also shared barriers stemming from the lived realities of participating. Community partners must consider their reputation and association with the project, as they will continue to live in the community beyond the project’s duration. This is coupled with the emotional labor required to participate in a project aimed at improving disparities in their community.

The participants also identified communication barriers with academic partners, specifically an overreliance on emails and lack of follow-up on decisions. They suggested institutionalizing formalized decision documentation and communication channels to enhance transparency and accountability. Community respondents recommended expanding streamlined, multimodal communication strategies that utilize visual displays of information and are translated into multiple languages. Finally, participants emphasized that not seeing academic partners in community-driven spaces was a barrier to engagement, making the partnership feel transactional rather than relational.

## Discussion

This study describes the participatory development and piloting of an equity-focused evaluation tool, including a survey and community storytelling, to address a gap in measuring the effectiveness of institutional advisory groups that span academic missions and applying the results [8,9]. The survey moves towards the goal of comparing across groups and exploration of associations between how groups function and their institutional impact [25,26]. While multiple tools exist to evaluate CE within research projects, including those we adapted from [4,6,7,27], our tool contributes to the literature in a number of important ways. First, we evaluated partnership processes and experiences within advisory groups across academic health or research center functions to assess community-centered outcomes. Second, our multi-methods participatory evaluation approach goes beyond measuring collaboration to building capacity for collaborative learning and adaptation, centering relational dynamics and power-sharing as evaluative endpoints. In doing so, this approach operationalizes equity as a continual, measurable practice – not just an intention – advancing the science of CE toward deeper accountability and impact.

In our pilot survey, we found that community-academic advisory groups within a NIH-funded center were generally positive about how the groups functioned and identified high levels of trust on the trust typology scale. As expected among these respondents with long-standing partnerships, the intensity of engagement related to enhanced perceptions of positive group functioning and the ability to manage conflict [28]. For this and marginal outcomes, results may also suggest reverse causality where those who perceive positive outcomes (e.g., ability to resolve conflicts, and the marginal associations with ability to reach consensus and promote decisions that affect communities) tend to engage more deeply with more frequent or persistent meeting attendance.

While quantitative measures provided a useful starting point, results suggested that the survey missed relational aspects of engagement. For example, higher community respondent scores related to pressure to conform to group decisions suggested the need to explore how power-sharing and decision-making function in practice and relate to outcomes. Respondents in storytelling sessions reiterated their overall satisfaction, but highlighted actionable challenges in power-sharing, decision-making, funding equity, and trust-building. For example, while survey responses suggested high levels of trust, storytelling sessions highlighted that trust is an ongoing process rather than a fixed outcome and pointed to specific actions, such as increasing meetings in community settings and community-responsive engagement strategies, that could be undertaken to improve trust, particularly in novice partnerships. Paired with qualitative and participatory methods, such as storytelling sessions, co-designed assessment tools offer a richer understanding of how partnerships function for those most affected. They also reveal gaps between institutional perceptions and community realities.

Additional outcomes suggest that participatory evaluation processes have utility. Participatory evaluation distributes power so that community members are not just being evaluated – they co-create the assessment itself [26]. This approach prioritizes participatory and qualitative methods to capture the lived experiences of both academic and community partners. Integrating structured survey data with open-ended discussions, the evaluation highlighted not just whether engagement was occurring, but how it was experienced. Community partners shared where inequities persist, how trust is built or broken, and what structures support or hinder meaningful participation. Addressing these barriers will allow advisory boards to be true spaces for shared governance, not just symbolic representation. By centering participatory evaluation as both a process and a product, this equity-centered evaluation model extends beyond conventional mixed methods to transformative engagement, making the evaluation itself an intervention that reshapes how knowledge is produced and utilized.

Participatory and qualitative approaches were central to producing meaningful, context-specific insights. The collaborative development of the survey tool – with input from both community and academic partners – functioned as both a process measure and an outcome in itself, reflecting the values of co-creation and shared ownership. Insights from storytelling sessions added necessary depth to the survey findings, revealing power dynamics, barriers to engagement, and strategies for more equitable collaboration. This mixed-methods design did more than triangulate results; it expanded the scope of what counted as evidence by incorporating experiential knowledge and community-defined indicators of success. These findings affirm that community-engaged analysis contributes to more actionable and trustworthy knowledge.

Limitations of the study include our small sample size within a single center. However, even given our small sample size and the fact that two of the groups were overrepresented, our findings captured differences in outcomes across groups with differing levels of experience, which is reassuring regarding the survey’s ability to capture variation. Our questions were largely drawn from the Engage for Equity CE Survey [8,29], which has been validated over a number of iterations of development. While our adaptation built from this prior work, and our CoP group reviewed and edited all items over multiple cycles for clarity and community comprehension, and to ensure that the questions reflected real concerns and experiences, we did not perform formal validity testing. Their input was crucial in identifying expected outcomes that aligned with community priorities and conceptualizations of impact. Furthermore, while individual items did show variability in responses, our scales for community-engaged group processes were largely positive, despite differing perceptions from the storytelling sessions. This may represent social desirability bias, though this seems unlikely in this anonymous survey given participants’ willingness to share concerns in a group storytelling session. Additional limitations include that clinical outcomes were not evaluated, as the center does not have a clinical component. Future research should validate the use of this tool across a larger set of advisory groups, inclusive of those with clinical focus.

For the qualitative portion of the toolkit, despite the small number of participants, the depth and consistency of participants’ reflections offer evidence of thematic saturation, a recognized marker of rigor in qualitative research [20]. Within a participatory and experiential evaluation, repeated patterns across diverse individuals – especially when drawn from structurally marginalized communities – can yield important insights. The convergence of lived experiences across multiple storytelling sessions in this evaluation highlights systemic dynamics not as isolated anecdotes, challenges dominant paradigms that overlook the analytic power of fewer, yet deeply engaged, voices and reinforces calls for methodological pluralism in equity-centered research [4,24].

Moving forward, the tool will be implemented annually across the institution, with results informing group process capacity-building and troubleshooting. Survey components will be reviewed across outcomes, with the process housed in the institution’s CTSA-supported CE team. This multi-method approach positions evaluation as a vehicle for learning and institutional transformation, guiding capacity-building efforts based on annual results.

This study demonstrates the value of participatory evaluation in institutional learning and transformation. By embedding lived experience into both the content and process of evaluation, equity becomes measurable and actionable. Future efforts should prioritize iterative, community-driven approaches that build institutional capacity, foster authentic collaboration, and move beyond inclusion toward true power-sharing.
